# Age-appropriate vaccination coverage and its determinants in children aged 12–36 months in Nepal: a national and subnational assessment

**DOI:** 10.1186/s12889-021-11841-2

**Published:** 2021-11-10

**Authors:** Santosh Kumar Rauniyar, Yoko Iwaki, Daisuke Yoneoka, Masahiro Hashizume, Shuhei Nomura

**Affiliations:** 1 Department of Global Health Policy, Graduate School of Medicine, The University of Tokyo, 7-3-1, Hongo, Bunkyo-ku, Tokyo, 113-0033 Japan; 2 SciREX Centre, National Graduate Institute for Policy Studies, Tokyo, Japan; 3 Graduate School of Public Health, St. Luke’s International University, Tokyo, Japan; 4 Department of Health Policy and Management, School of Medicine, Keio University, Tokyo, Japan

**Keywords:** Delay, Timeliness, Vaccine coverage, Vaccination, Immunization

## Abstract

**Background:**

Vaccination is one of the effective ways to develop immunity against potential life-threatening diseases in children in early age. This study is focused on analysing the age-appropriate vaccination coverage at national and subnational levels and identify the factors associated with age-appropriate coverage in Nepal.

**Methods:**

460 children aged 12–36 months were included in the study. The data was obtained from Nepal Demographic and Health Survey (NDHS) 2016–17. Age-appropriate coverage of Bacillus Calmette-Guerin vaccine (BCG), oral polio vaccine (OPV) doses 1–3, pentavalent vaccine (PE) doses 1–3, and first dose of measles, mumps, and rubella vaccine (MMR) were estimated using Kaplan Meier method. Multilevel logistic regression with random intercept was used to identify the factors associated with age-appropriate vaccination.

**Results:**

The crude coverage of the vaccines included in the study ranged from 91.5% (95% CI, 88.5–93.7) for PE3 to 97.8% (95.8–98.7) for BCG. Although the crude coverage of all the vaccines was above 90%, the age-appropriate coverage was significantly low, ranging from 41.5% (36.5–46.6) for PE3 to 73.9% (69.2–78.1) for PE1. Furthermore, high disparity in timely vaccination coverage was observed at regional level. Compared to the age-appropriate vaccination coverage in other provinces, Province 2 had the lowest coverage of all, followed by that in Province 6. The timeliness of vaccination was significantly associated with subnational regions i.e., provinces and the season of childbirth.

**Conclusion:**

Although the immunization program in Nepal has achieved the target of 90% crude coverage of all the childhood vaccines, the age-appropriate coverage is significantly low which undermines the effectiveness of the vaccines administered. Thus, along with crude coverage, timeliness of the vaccines administered should be taken into consideration and thoroughly monitored at national and subnational levels. Provincial government should formulate tailored strategies to ensure the timely administration of the childhood vaccines.

**Supplementary Information:**

The online version contains supplementary material available at 10.1186/s12889-021-11841-2.

## Background

Vaccination is one of the effective ways to develop immunity against potential life-threatening diseases in children in early age [1]. For instance, diseases such as polio and diphtheria are becoming rare in many countries around the world due to effective and timely vaccination [2, 3]. To reduce the risk of getting exposed to vaccine preventable diseases and to increase immunization coverage of basic childhood vaccines, World Health Organization (WHO) initiated the Expanded Programme on Immunization (EPI) in 1974. Remarkable progress has been made worldwide since the implementation of EPI [4, 5]. For instance, over the last decade, more than 1 billion children have been vaccinated and an estimated 2 to 3 million death has been averted through immunization worldwide [5, 6]. However, at the same time, nearly 20 million children still face insufficient access to vaccines globally [7–9]. The resurgence of vaccine preventable diseases (VPD) such as measles in Mongolia, USA and in other countries has emphasized that not only coverage rate but also timeliness of the vaccines administered is important to ensure effective immunization [10–13].

In Nepal, the National Immunization Program (NIP) was implemented in 1979 with the objectives to increase immunization coverage and control the vaccine-preventable diseases [14]. The immunization programme has performed well and has been considered success in recent years. In 2017, the crude vaccination coverage for most of the vaccines was reported above 80% [15]. However, the increasing cases of measles and high prevalence of tuberculosis in Nepal in recent years has posed an important question on the effectiveness of the immunization program [16, 17]. Currently, the surveillance report on immunization by WHO emphasized that the immunization program is solely focused on attaining high coverage rate while neglecting the timeliness of the vaccines administered [15]. Delay in immunizations may cause outbreaks of infectious disease since vaccines delivered outside the immunization schedule leave temporal gaps in immunity in which children are vulnerable to infections [18]. Hence, to realize the full benefits of immunization program, it is important to consider timely administration of the vaccines along with the high coverage rate. However, there are no studies been conducted at national level to access the timeliness of childhood vaccines in Nepal. Thus, this is the first study aimed to analyse the age-appropriate vaccination coverage at national and subnational levels and to identify the factors associated (compliance) with age-appropriate vaccination in Nepal.

## Method

### Data source

We used recently available data from Nepal Demographic and Health Survey, (NDHS) 2016–17. NDHS is a nationally representative population-based cross-sectional household surveys that included information about maternal and child health. Data were collected from June 2016 to January 2017. Out of 11,472 occupied households 11,203 were interviewed with response rate of 99.0%. The survey used multistage stratified cluster sampling design method to collect the data. The questionnaire for children under five was administered to mothers (or caretakers) of the children through women’s questionnaire. In total 6091 children under five years were selected with the response rate of 98.6%. The details of sampling methods and questionnaires are described elsewhere [19].

### Study population

Initially, 975 children aged 12–36 months were included in the study. Out of 975 children, 69 of them who did not have mother or child health books or vaccination cards (which are official written records of vaccination history provided by Government of Nepal [14]) were excluded. Furthermore, 446 children those who lost or no longer have vaccination card were excluded from the study. For the final analyses, 460 children were included in the study who had complete information about vaccine administration date.

### Vaccines

The Vaccines assessed in this study were Bacillus Calmette-Guerin vaccine (BCG); Oral polio, doses 1–3 (OPV1, OPV2, and OPV3); Pentavalent vaccine (DTP-Diphtheria, Tetanus, and Pertussis vaccine; Hep B Hepatitis B vaccine; Hib-Hemophilus influenzae type b vaccine), doses 1–3 (PE1, PE2, and PE3); and Measles, mumps, and rubella vaccine first dose (MMR1) (Table 1).

**Table 1:** The national immunization schedule, Nepal [14]

Name of vaccines	At birth (at 0–30 day)	6 weeks of age (at 42–72 day)	10 weeks of age (at 70–100 day)	14 weeks of age (at 91–121 day)	9 months of age (at 274–304 day)
BCG	BCG0				
OPV		OPV1	OPV2	OPV3	
Pentavalent (DPT, Hep B, and Hib)		Penta1	Penta2	Penta3	
MMR					MMR 1

BCG-Bacillus Calmette-Guerin vaccine; OPV-Oral Polio vaccine; DTP-Diphtheria, Tetanus, and Pertussis vaccine; Hep B Hepatitis B vaccine; Hib-Hemophilus influenzae type b vaccine; MMR-Measles, Mumps, and Rubella vaccine; numbers indicate a dose order.

#### Crude and age-appropriate vaccine coverage

The proportion of children who received the routine vaccines regardless of the age at which they received the vaccine was considered as crude vaccine coverage

The age-appropriate vaccination was defined as children who received a vaccine dose within the recommended age according to the immunization schedule of National immunization Programme (NIP) Nepal, (Table 1) [14], plus 30 days grace period after the due date. The grace period for age-appropriate vaccination was decided based on previous studies [20, 21]. The administration date of the vaccines was calculated by subtracting the date of birth from the date of the vaccination. Children receiving the vaccines after the recommended age-range were considered to have received delayed vaccination. Vaccines administered before the recommended age-range was defined as early vaccination. Children who had been marked as not given vaccines or marked as given vaccines, but no date found on the mother and child health book or vaccination card were considered as children not vaccinated.

### Statistical analysis

The proportion of crude and age-appropriate vaccine coverage with 95% confidence interval (CI) were calculated for each vaccine dose at national and regional levels. To analyse the timeliness of the vaccines administered according to the immunization schedule of NIP Nepal, we used Kaplan-Meier product limit method. Due to the multi-stage sampling method, all the analyses were adjusted to the sampling weight.

Next, we used multivariate logistic regression to investigate the association between age-appropriate vaccination and socioeconomic variables, as well as characteristics of the children and their parents, including gender of the children, mothers’ age, mothers’ education, socio-economic status of households, religion of household heads, ethnicity, area of residence, mothers’ occupation, and season of childbirth. To select the covariates, we used the backward stepwise variable selection method with cut-off level at *p* < 0.05. The regression models included random effects at cluster levels to control for correlation among different clusters. The restricted maximum likelihood method was used to estimate the regression parameters. *P* value < 0.05 was considered for statistical significance. STATA/SE 15.1 and R programming were used to analyse the data and create geospatial mapping.

We used the STROBE cross-sectional reporting guidelines, the standard guidelines to report cross-sectional study [22].

## Results

### Sample characteristics

For the analyses, we incorporated data of 460 eligible children aged between 12 and 36 months from the total sample size 6091 children aged 0–59 months (975 aged between 12 and 36 months) included in NDHS. 54.6% (*n* = 251) children included in the study were male.54.4% (250) children had mothers aged between 25 and 44 years and 56.2% (258) children had mothers having secondary school or higher education background (Table 2). Out of total sample population, 46.2% (213) belonged to Dalit and Janjati ethnicity and 44.5% (205) lived in rural areas. 70.4% of mothers had antenatal visit more than three times during the pregnancy. (Table 2). Among the 515 children who were excluded from the study 49.7% (259) belonged to the households having poorest and poorer wealth quintile. 38.5% (201) children’s mothers had no education. 93.5% (487) children’s mother were aged between 15 and 34 years. In addition, 27.1% (142) children were from Province 2.

**Table 2:** Sample characteristic of 460 children aged 12–36 months, in Nepal, 2016

Variables	Number	Proportion (%)
Gender		
Male	251	54.6
Female	209	45.4
Mother’s age		
15–24	210	45.6
25–34	218	47.4
35–44	32	7.0
Mother’s education		
No formal education	104	22.6
Primary level education	98	21.2
Secondary level education	172	37.5
Higher education	86	18.7
Ethnicity		
Bhrahmin/Chettri	142	30.8
Dalit and Janjati	213	46.2
Newar	18	3.9
Muslim	18	4.1
Others	69	15.0
Area of residence		
Urban	255	55.5
Rural	205	44.5
Province		
Province 1	72	15.6
Province 2	78	16.9
Province 3	83	18.2
Province 4	57	12.4
Province 5	102	22.1
Province 6	28	5.9
Province 7	40	8.9
Season of childbirth		
Winter	139	30.2
Spring	120	26.1
Summer	100	21.7
Autumn	101	22.0
ANC visits		
Not visited	15	3.4
Visited once	13	2.9
Visited twice	22	4.6
Visited 3 times	47	10.1
Visited more than 3 times	324	70.4
Missing	39	8.6

ANC-Antenatal care. The given sample size is adjusted to the survey sample weight.

### Crude and age-appropriate vaccine coverage

The crude vaccination coverage ranged from 91.5% (95% CI, 88.5–93.7) for PE3 to 97.8% (95.8–98.7) for BCG. Although the crude coverage of all the vaccines was above 90%, the age-appropriate coverage was significantly low ranging from 41.5% (36.5–46.6) for PE3 to 73.9% (69.2–78.1) for PE1. (Table 3)

**Table 3:** Crude and age-appropriate vaccination coverage in Nepal (*n* = 460)

Vaccines	Crude coverage	Age-appropriate coverage	Early vaccination	Delayed vaccination
	Proportion (95% CI)	Proportion (95% CI)	Proportion, 95% CI	Proportion, 95% CI
BCG	97.77 (95.85–98.75)	54.58 (49.33–59.73)	–	45.41 (40.26–50.67)
OPV1	96.19 (94.00–97.60)	73.13 (68.31–77.46)	5.03 (3.20–7.84)	21.83 (17.86–26.39)
OPV2	95.56 (93.23–97.10)	60.13 (54.97–65.07)	1.82 (0.85–3.86)	38.05 (33.17–43.18)
OPV3	94.38 (91.86–96.16)	41.74 (36.72–46.93)	0.36 (0.06–2.00)	57.91 (52.71–62.94)
Penta1	97.69 (95.83–98.73)	73.94 (69.24–78.14)	4.70 (2.97–7.38)	21.35 (17.49–25.80)
Penta2	97.21 (95.22–98.38)	60.61 (55.56–65.44)	1.56 (0.69–3.46)	37.83 (33.05–42.86)
Penta3	91.49 (88.55–93.73)	41.48 (36.54–46.59)	–	58.38 (53.26–63.31)
MMR1	96.15 (93.94–97.57)	53.83 (48.53–59.04)	11.00 (8.10–14.77)	35.17 (30.30–40.38)

CI-Confidence interval; BCG-Bacillus Calmette-Guerin vaccine; OPV-Oral Polio vaccine; DTP-Diphtheria, Tetanus, and Pertussis vaccine; Hib-*Haemophilus influenzae* type b vaccine; MMR-Measles, Mumps, and Rubella vaccine; numbers indicate a dose order

**Fig. 1:**
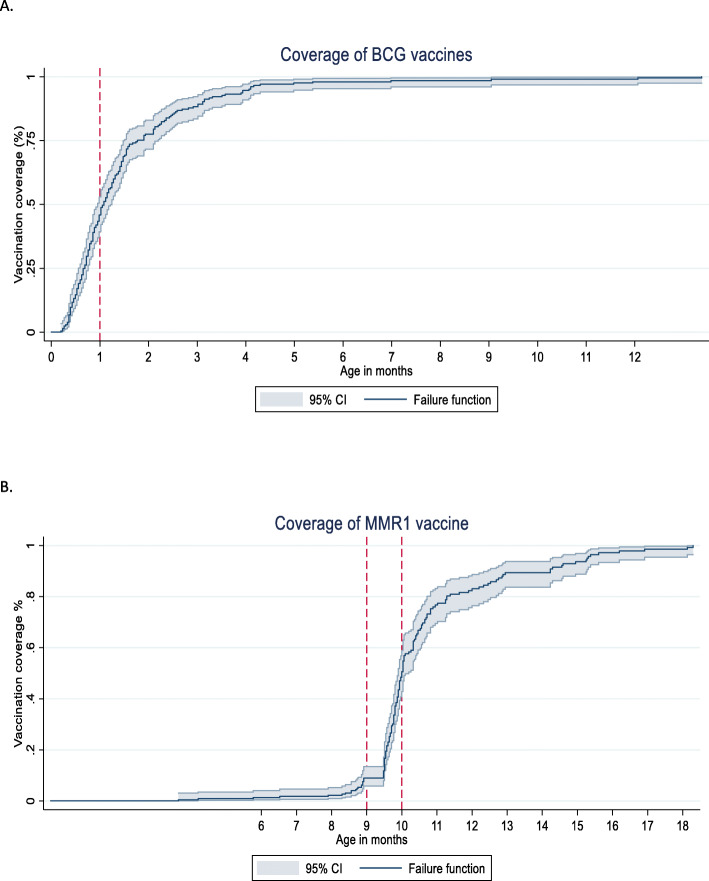
Age-appropriate coverage of BCG and MMR1 vaccines at national level in Nepal, 2016. **A**. A Bacillus-Calmette-Guerin (BCG) vaccine. **B**. Measles, Mumps, and Rubella vaccine, 1st dose (MMR1). Note: BCG-Bacillus Calmette-Guerin vaccine; MMR1-Measles, Mumps, and Rubella vaccine, 1st dose. CI-Confidence interval. * The red lines in the figure indicate the age-appropriate time range for the vaccine to be administered

**Fig. 2:**
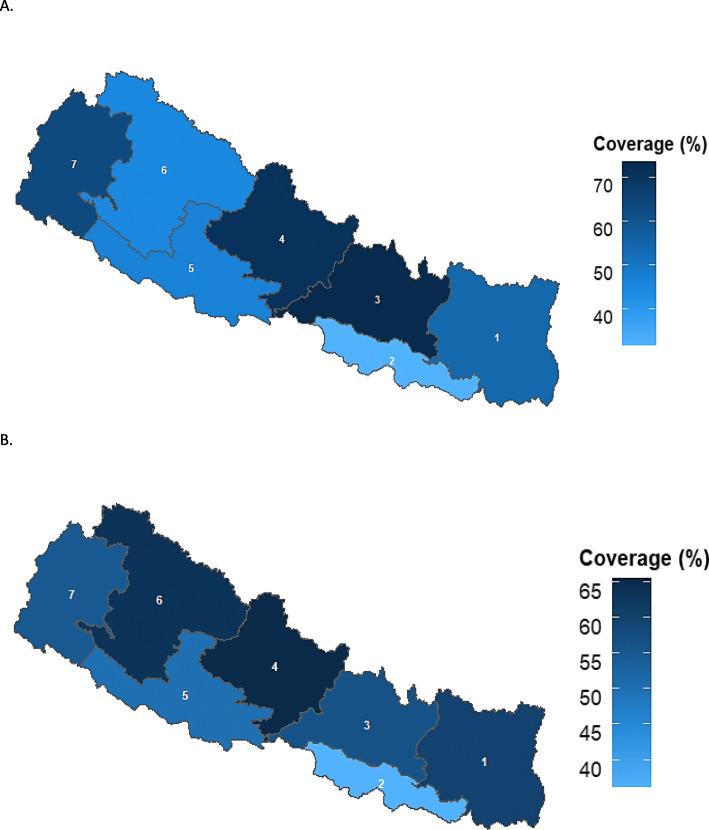
Age-appropriate coverage of BCG and MMR vaccines l at regional level in Nepal, 2016. **A**. Bacillus-Calmette-Guerin (BCG) vaccine. **B**. Measles, Mumps, and Rubella vaccine (MMR) 1st dose. Note: 1,2,3,4,5,6 and 7 are the respective provinces in Nepal. Note: BCG-Bacillus Calmette-Guerin vaccine; MMR1-Measles, Mumps, and Rubella vaccine, 1st dose. CI-Confidence interval

Figures 1 and 2 show the age-appropriate coverage of BCG, vaccine at national and regional level. As shown in Fig. 1 and Tables 3, 54.6% (95% CI, 49.3–59.7) of the children were vaccinated for BCG within the recommended age-range. At regional level, Province 3 has the highest age-appropriate coverage that was 73.3% (59.6–83.6) followed by Province 4 that was 70.7% (56.8–81.7). Province 2 had the lowest age-appropriate BCG coverage which was 31.9% (20.4–46.0) followed by Province 6, 44.9% (25.5–65.9) (Fig. 2).

For the MMR1 vaccine, 53.8% (95% CI, 48.5–59.0) of the children were vaccinated within the recommended age-range (Fig. 1 and Table 3). The proportion of delayed vaccination was 31.2% (30.3–40.4) (Table 3). At regional level, Province 4 has the highest age-appropriate coverage for MMR1 vaccine 65.4% (51.1–77.4) followed by Province 6, 63.3% (39.3–82.1). Similar to OPV and PE vaccine, Province 2 has the lowest age-appropriate coverage for MMR1, 36.1% (24.3–49.9) (Fig. 2).

**Fig. 3:**
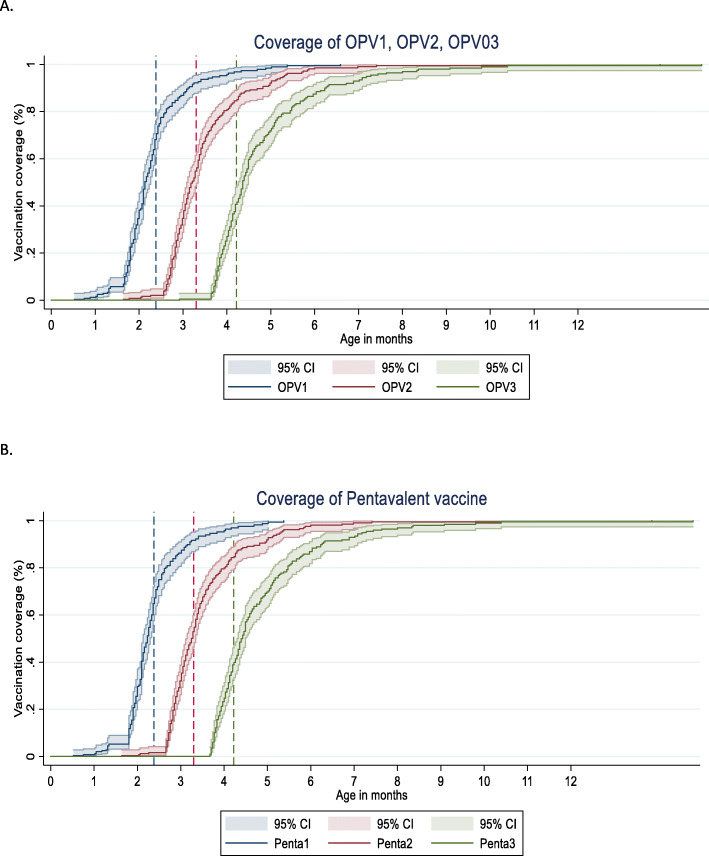
Age-appropriate coverage of OPV and PE vaccines at national level in Nepal, 2016. **A**. Oral polio vaccine (OPV) 1–3 doses. **B**. Pentavalent vaccine doses (Penta) 1–3 doses. Note: OPV-Oral Polio vaccine; Penta1, Penta2, Penta3- Pentavalent vaccines doses 1–3 (DTP-Diphtheria, Tetanus, and Pertussis vaccine; Hep B- Hepatitis B vaccine; Hib-Hemophilus influenzae type b vaccine); CI-Confidence interval. * The blue, red, and green lines in the figure indicate the age-appropriate time range for the vaccine to be administered

**Fig. 4:**
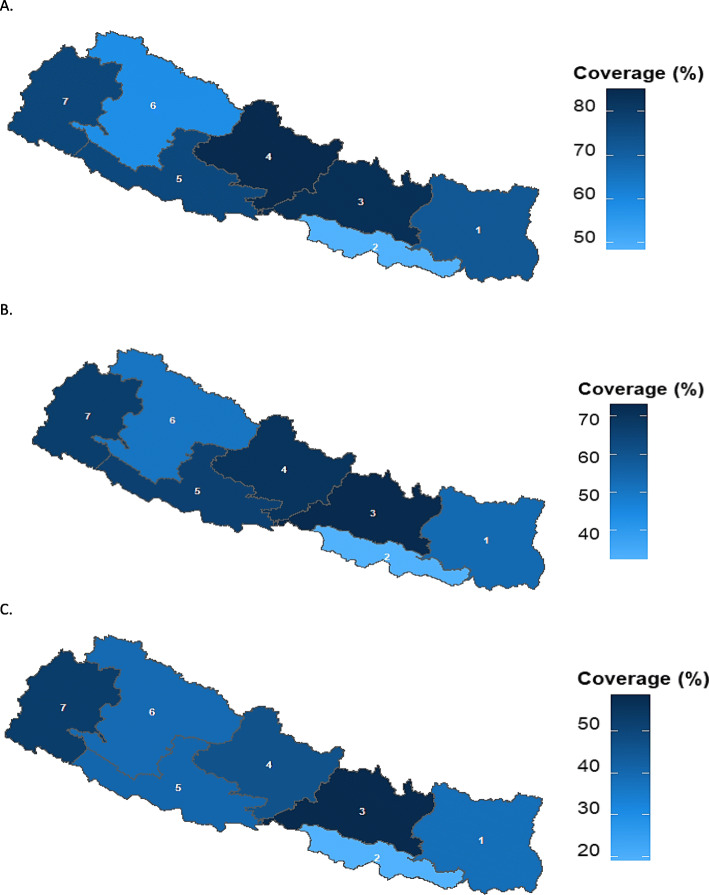
Age-appropriate coverage of Oral polio vaccines doses 1–3 at regional level in Nepal, 2016. **A**. Oral polio vaccine 1st dose (OPV1). **B**. Oral polio vaccine 2nd dose (OPV2). C. Oral polio vaccine 3rd dose (OPV3). Note: 1,2,3,4,5,6 and 7 are the respective provinces in Nepal

Figures 3 and 4 shows the age-appropriate coverage of OPV1-OPV3 (OPV1, OPV2, and OPV3) vaccines received by children over time at national and regional levels. As shown in Fig. 3 and Table 3, for the OPV1 vaccine 73.13% (95% CI, 68.3–77.5) of the children received it at recommended age. For OPV second and third doses (OPV2 and OPV3) these number were 60.1% (54.9–65.1) and 41.7% (36.7–46.9), respectively. The proportions of delayed vaccination for OPV1, OPV2, and OPV3 were 21.8% (17.9–26.4), 38.1% (33.2–43.2), and 57.9% (52.7–62.9) respectively (Table 3). At regional level, Province 4 has the highest age-appropriate coverage of OPV1, and OPV2 while Province 3 has highest age-appropriate coverage of OPV3 vaccines. Province 2 has the lowest age-appropriate coverage for all doses of OPV vaccine (Fig. 4).

**Fig. 5:**
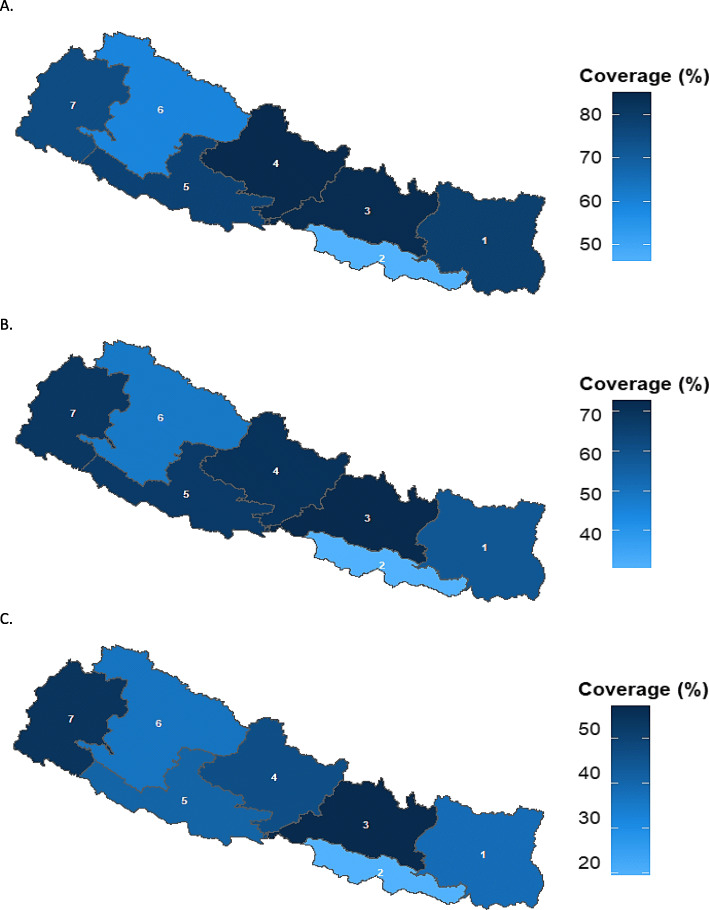
Age-appropriate coverage of Pentavalent vaccines doses 1–3 at regional level in Nepal, 2016. **A**. Pentavalent 1st dose (Penta1). **B**. Pentavalent 2nd dose (Penta2). **C**. Pentavalent 3rd dose (Penta3). Note: 1,2,3,4,5,6 and 7 are the respective provinces in Nepal

Figures 3 and 5 present children who received age-appropriate PE1-PE3 (PE1, PE2, and PE3) vaccines over time at national and regional levels. For PE1-PE3 vaccines, 73.9% (95% CI, 69.2–78.1), 60.6% (55.6–65.4), and 41.2% (36.5–46.6) children were vaccinated within the recommended age-range respectively (Table 3). The proportions of delayed vaccination for PE1, PE2, and PE3 were 21.4% (17.5–25.8), 37.8% (33.1.7–42.9), and 58.4% (53.3–63.3). At regional level, Province 4 has the highest age-appropriate coverage for the first dose of pentavalent vaccine; 84.9 (72.4–92.3) followed by Province 3; 84.3 (72.9–91.5). Province 3 had highest age-appropriate coverage of second and third doses of pentavalent vaccine; 72.5% (60.0–82.2), and 57.0 (44.1–68.9) respectively. Province 2 has the lowest age-appropriate coverage for all the doses of PE vaccine among all (Fig. 5).

### Factors associated (compliance) with age-appropriate vaccination of BCG, OPV1-OPV3, PE1-PE3 and MMR vaccines

The significant results from multilevel logistic regression models are shown in Table 4 and Table 5. The regression analysis showed that the children born in spring and winter had significantly higher odds of receiving age-appropriate BCG vaccines (odds ratio [OR], 2.34, 95% CI, 1.21–4.54) for spring, (3.86, 1.83–8.13) for winter compared to those born in summer. Children in Province 2 and Province 6 have significantly lower odds of receiving timely vaccination for BCG, OPV1, OPV2, PE1, and PE2 compared to children in other provinces. (Table 4 and Table 5). Other factors did not show significant association.

**Table 4:** Multilevel logistic regression results for BCG and MMR1 vaccine (n = 460)

Variables	Odds ratio (95% CI) for age-appropriate vaccination	
	BCG	MMR1
Province		
Province 3	1.00 (ref [1])	1.00 (ref)
Province 1	0.53 (0.19–1.47)	0.66 (0.21–2.07)
Province 2	0.21 (0.07–0.67)**	1.42 (0.40–5.05)
Province 4	0.82 (0.29–2.31)	0.55 (0.17–1.77)
Province 5	0.31 (0.11–0.85)*	0.90 (0.29–2.76)
Province 6	0.28 (0.09–0.88)*	0.57 (0.17–1.90)
Province 7	0.70 (0.24–2.05)	0.86 (0.27–2.71)
Season of childbirth		
Summer	1.00 (ref)	1.00 (ref)
Spring	2.34 (1.21–4.54)*	1.21 (0.56–2.60)
Autumn	1.63 (0.81–3.27)	1.46 (0.66–3.23)
Winter	3.86 (1.83–8.13)***	1.84 (0.81–4.19)

CI- Confidence interval; ref-reference; BCG-Bacillus Calmette-Guerin vaccine; MMR-Measles, Mumps, and Rubella vaccine; * p < 0.05; ** *p* < 0.01; *p* < 0.001; Random effect at PSU level was incorporated to account for survey design

**Table 5:** Multilevel logistic regression results for OPV1-OPV3 and PE1-PE3 (n = 460)

Variables	Odds ratio (95% CI) for age-appropriate vaccination					
	OPV1	OPV2	OPV3	PE1	PE2	PE3
Province						
Province 3	1.00 (ref)	1.00 (ref)	1.00 (ref)	1.00 (ref)	1.00 (ref)	1.00 (ref)
Province 1	0.72 (0.20–2.54)	0.34 (0.10–1.10)	0.45 (0.17–1.15)	0.79 (0.27–2.30)	0.44 (0.14–1.31)	0.60 (0.28–1.27)
Province 2	0.23 (0.07–0.82)*	0.14 (0.04–0.53)**	0.21 (0.07–0.66)**	0.26 (0.08–0.91)*	0.17 (0.05–0.60)**	0.26 (0.11–0.62)*
Province 4	1.27 (0.36–4.55)	0.73 (0.20–2.62)	0.63 (0.26–1.54)	2.04 (0.66–6.31)	1.02 (0.31–3.31)	1.09 (0.56–2.13)
Province 5	0.95 (0.30–3.00)	0.69 (0.21–2.26)	0.52 (0.22–1.22)	1.29 (0.45–3.66)	1.11 (0.36–3.41)	0.86 (0.48–1.53)
Province 6	0.30 (0.09–0.97)*	0.25 (0.07–0.87)*	0.46 (0.15–1.43)	0.28 (0.09–0.89)*	0.27 (0.08–0.88)*	0.57 (0.22–1.49)
Province 7	0.75 (0.23–2.43)	0.58 (0.17–2.02)	0.87 (0.34–2.20)	0.92 (0.30–2.75)	0.79 (0.25–2.50)	1.22 (0.61–2.44)
Wealth quintile						
Poorest	1.00	1.00	1.00	1.00	1.00	1.00
Poorer	1.58 (0.69–3.64)	0.87 (0.40–1.89)	0.91 (0.46–1.83)	1.13 (0.58–2.18)	0.83 (0.41–1.66)	0.99 (0.54–1.81)
Middle	0.91 (0.40–2.08)	0.97 (0.43–2.18)	0.76 (0.35–1.63)	0.63 (0.30–1.32)	0.91 (0.43–1.92)	0.74 (0.35–1.58)
Richer	2.19 (0.78–6.09)	1.11 (0.44–2.81)	0.85 (0.37–1.92)	1.25 (0.47–3.29)	0.97 (0.43–2.22)	0.81 (0.36–1.80)
Richest	1.17 (0.38–3.63)	1.10 (0.39–3.11)	1.39 (0.62–3.16)	0.74 (0.25–2.14)	0.80 (0.29–2.17)	1.07 (0.49–2.32)
Season of childbirth						
Summer	1.00 (ref)	1.00 (ref)	1.00 (ref)	1.00 (ref)	1.00 (ref)	1.00 (ref)
Spring	0.61 (0.29–1.30)	0.55 (0.26–1.17)	1.24 (0.63–2.47)	0.83 (0.34–2.00)	0.60 (0.30–1.17)	1.15 (0.55–2.41)
Autumn	0.81 (0.35–1.89)	0.86 (0.38–1.95)	1.63 (0.80–3.34)	0.96 (0.46–2.00)	0.89 (0.44–1.81)	1.54 (0.81–2.92)
Winter	0.72 (0.30–1.70)	0.91 (0.43–1.92)	1.82 (0.98–3.39)	0.83 (0.42–1.66)	0.91 (0.47–1.75)	1.63 (0.93–2.85)

CI-Confidence interval; ref-reference; OPV-Oral Polio vaccine; DTP-Diphtheria, Tetanus, and Pertussis vaccine; Hep B-Hepatitis B vaccine; Hib-Hemophilus influenzae type b vaccine; numbers indicate a dose order; * p < 0.05; ** p < 0.01; p < 0.001; Random effect at PSU level was incorporated to account for survey design

## Discussion

Full benefits of vaccination could be attained through high coverage and timely administration. Nepal has already met the immunization target set by WHO to attain 90% coverage for most of the childhood vaccines. According to WHO/ UNICEF estimates of immunization coverage report 2019, the crude coverage for most of the vaccines were above 90% in Nepal. The 2018/19 annual report provided by the Health Ministry of Nepal reported that the crude coverage for some vaccines such as BCG was 92% [15]. However, according to our study findings, the age-appropriate coverage of BCG vaccine was below 60% at national level.

Although immunization program has been considered successful in Nepal with target coverage being met [23], low age-appropriate coverage of these vaccines remains a big issue. Several studies conducted in similar settings in different countries estimated low age-appropriate coverage of childhood vaccines [12, 20, 24–27]. The recent increase in number of measles and tuberculosis cases in Nepal could be attributed to untimely vaccination in Nepal [17, 28]. The reasons behind the low age-appropriate vaccines coverage rate might include lack of awareness about the immunization schedule, hard to access health care facilities, reluctancy in administering vaccines, hesitancy of parents regarding vaccination, insufficient infrastructure to transport and store the vaccine in hard to reach areas, and occurrence of unusual events such as natural disaster, disease outbreak (pandemic situation) [20, 26, 27, 29–33].

The huge earthquake of 2015 in Nepal significantly affected the healthcare services in different provinces throughout the country [34]. Our study was conducted after the earthquake. Therefore, it is highly possible that the 2015 earthquake could have a significant effect on health care facilities leading to delay in vaccination and resulting in low age-appropriate coverage. Similarly, it could be inferenced that Covid19 outbreak would affect the vaccination program in different regions and would increase the risk to the resurgence of VPD [35]. Therefore, to cope with the unforeseen circumstances such as natural disasters and disease outbreaks, the central government along with the local government should focus on capacity building for disaster preparedness, improve basic infrastructure, mostly in hard to reach areas, and strengthen community healthcare facilities. Furthermore, provincial governments should focus on planning and setting framework based on local situation at regional levels.

In this study we found that along with low age-appropriate coverage, the timely coverage of later doses of vaccines subsequently declines compared to the former doses. For instance, timely coverage of second and third doses of OPV and PE vaccines significantly decreases as compared to its respective first dose. This result is similar to those found in the neighbouring countries such as Bangladesh and Pakistan [26, 36]. One of the possible explanations for this could be increase in workload for mothers and increase in domestic activities while a child become older. Another explanation could be the adverse events such as fever, pain or swelling on the injection site, following the prior doses that would restrain mothers for the next appointment [37, 38]. Furthermore, parents’ perception that the later doses are not as important as the first dose, and reluctancy to follow up could explain the existing low age-appropriate coverage for later doses [29, 38].

At regional level, high disparity was observed in age-appropriate vaccination coverage. In Province 2 and Province 6, timely coverage of the vaccines included in this studied  was lower compared to that in other regions. Though, geographically Province 2 is easily accessible, the low vaccination coverage could be due to low compliance rate, low literacy rate, hesitancy towards vaccination, lack of knowledge about the immunization program, lack of proper health care infrastructure in rural areas, and other cultural barriers [39–41]. Use of mobile phone/smart phone (mhealth) to improve the knowledge and awareness about vaccination and immunization schedule could be an effective way [42, 43]. In case of Province 6, low age-appropriate coverage could be due to hard to reach terrain, lack of awareness about the immunization schedule, lack of sufficient infrastructure such as transportation and storage facilities, and lack of human resource in health sector [32, 33]. Use of drones technology to transport vaccines in hard to reach areas could solve the problem in these regions [44]. Province 3 had the highest coverage of almost all the vaccines as it is the central region that includes capital city Kathmandu, and most of the areas in this province are developed [19, 41].

As highlighted in the previous study [20], the analysis of vaccine data using the DHS has several limitations. First, only children who had vaccination records in the mother and child health book (the vaccination card) were included. Due the exclusion of children who did not have the vaccination card, the sample size has reduced to 460 which is not a large sample size for this study hence posing a limitation to the study. In addition, exclusion of children without vaccination records might lead to overestimation of the vaccination coverage and timeliness if these children were less likely to receive adequate vaccinations. Children who were excluded from our analyses due to missing data on vaccination were more likely to be from the poorest household as compared with those included in the study. Second, age-appropriate vaccination coverage among children can be influenced by many other factors, including those related to access to health care services, knowledge, attitudes, and practices of parents and providers. The variables investigated in this study were limited to those available in DHS. Third, due to significant missing data and long administration period (between 4 to 6 years of age) of second dose of MMR vaccines we could not include it in the study. Finally, both early and delayed vaccinations were analyzed as a single category. Investigation of each of these types of untimely vaccinations is a topic for future studies.

## Conclusion

This is the first national level study conducted in Nepal focusing on the timeliness of childhood vaccination. Our study showed that although the crude coverage of childhood vaccines is above 90%, the age-appropriate coverage of these vaccines is significantly low at national and subnational levels. The national immunization program is solely focused on attaining high crude coverage while neglecting the importance of timeliness of the vaccines administered. The significantly low age-appropriate coverage of all the childhood vaccines at national and subnational level emphasizes the importance of formulating effective policies at national and subnational levels to improve the age-appropriate coverage rate. Increased focus on promoting awareness about the immunization schedule in several regions, particularly, in the provinces with significantly low age-appropriate coverage (Province 2 and Province 6) is of prime importance.

## Supplementary Information

**Additional file 1.**

## Data Availability

The datasets analyzed during the study are available in the Demographic and Health Surveys, DHS repository, https://dhsprogram.com/data/dataset/Nepal_Standard-DHS_2016.cfm?flag=0

## Abbreviations

BCG: Bacillus Calmette-Guerin Vaccine
CI: Confidence interval
DPT: Diphtheria, Tetanus and Pertussis Vaccine
EPI: Expanded Programme on Immunization
Hep B: Hepatitis B Vaccine
Hib: Hemophilus Influenza Type b Vaccine
MMR: Measles, Mumps, and Rubella Vaccine
NDHS: Nepal Demographic and Health Surveys
NIP: National Immunization Program
OPV: Oral polio Vaccine
PE: Pentavalent Vaccine
VPD: Vaccine Preventable Diseases
WHO: World Health Organization
