# Supplementary material for: Age-appropriate vaccination coverage and its determinants in children aged 12–36 months in Nepal: a national and subnational assessment
Source: BMC Public Health. 2021 Nov 10;21:2063. doi: 10.1186/s12889-021-11841-2 (PMC8582094; doi:10.1186/s12889-021-11841-2)
Supplement: Supplementary file 1 — Additional file 1. [file 12889_2021_11841_MOESM1_ESM.docx]

**Supplementary Figure 1:** Age-appropriate coverage of childhood vaccines at provincial level in Nepal

**Supplementary Table 1:** Pair-wise correlation coefficient among independent variables.

**Supplementary Table 2:** Comparison of children aged 12-36 months included and excluded in the study.

**Supplementary Table 3:** Immunization target and the coverage reported by Ministry of Health Nepal in 2016- 17.

**Supplementary Material:** Detail method of data collection in Nepal

**Supplementary Figure 1. Age-appropriate coverage of childhood vaccines at provincial level in Nepal**


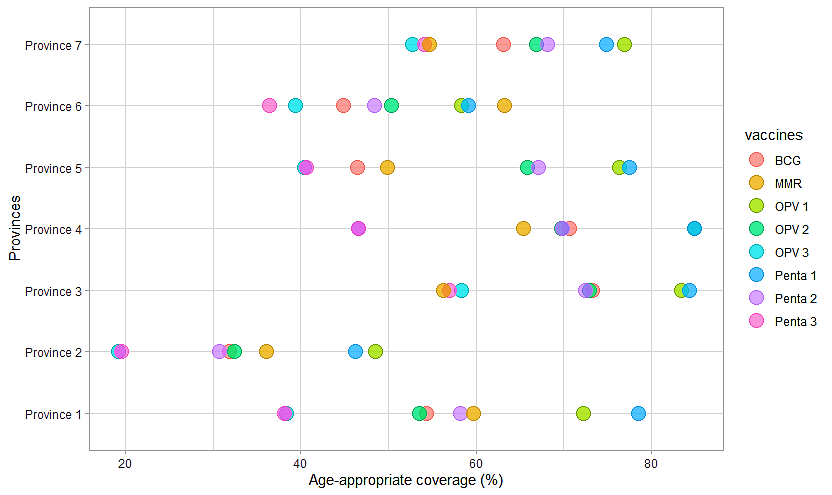


**Supplementary Table 1. Pair-wise correlation coefficient among independent variables**

| Variables | (mother’s age) | (education) | (Ethnicity) | (Residence) | (Province) | (Season of childbirth) | (Wealth quintile) | (Gender) | |  |
| --- | --- | --- | --- | --- | --- | --- | --- | --- | --- | --- |
| Mother’s age | 1.000 |  |  |  |  |  |  | |  | |
| Education | -0.184 | 1.000 |  |  |  |  |  | |  | |
| Ethnicity | -0.082 | -0.238 | 1.000 |  |  |  |  | |  | |
| Residence (Urban/rural) | 0.044 | -0.170 | 0.128 | 1.000 |  |  |  | |  | |
| Province | -0.088 | 0.004 | -0.318 | -0.048 | 1.000 |  |  | |  | |
| Season of childbirth | -0.049 | 0.025 | -0.034 | 0.069 | -0.029 | 1.000 |  | |  | |
| Wealth quintile | 0.037 | 0.327 | 0.255 | 0.077 | -0.261 | 0.003 | 1.000 | |  | |
| Gender | -0.093 | -0.003 | 0.007 | -0.099 | 0.009 | 0.014 | -0.029 | | 1.000 | |
|  | | | | | | | | | | |

**Supplementary Table 2: Comparison of children aged 12-36 months included and excluded in the study.**

| Variables | Children included in the study ( n = 460) | | Children excluded from the study (n = 515) | |
| --- | --- | --- | --- | --- |
|  | Number | Proportion (%) | Number | Proportion (%) |
| Gender |  |  |  |  |
| Male | 251 | 54.6 | 266 | 51.6 |
| Female | 209 | 45.4 | 249 | 48.7 |
| Mother's age |  |  |  |  |
| 15-24 | 210 | 45.6 | 247 | 48.1 |
| 25-34 | 218 | 47.4 | 235 | 45.6 |
| 35-44 | 32 | 7.0 | 33 | 6.3 |
| Mother's education |  |  |  |  |
| No formal education | 104 | 22.6 | 219 | 42.6 |
| Primary level education | 98 | 21.2 | 99 | 19.1 |
| Secondary level education | 172 | 37.5 | 136 | 26.3 |
| Higher education | 86 | 18.7 | 62 | 12.0 |
| Ethnicity |  |  |  |  |
| Bhrahmin/Chettri | 142 | 30.8 | 119 | 23.0 |
| Dalit and Janjati | 213 | 46.2 | 218 | 42.3 |
| Newar | 18 | 3.9 | 7 | 1.3 |
| Muslim | 18 | 4.1 | 40 | 7.8 |
| Others | 69 | 15.0 | 131 | 25.6 |
| Area of residence |  |  |  |  |
| Urban | 255 | 55.5 | 261 | 50.7 |
| Rural | 205 | 44.5 | 254 | 49.3 |
| Province |  |  |  |  |
| Province 1 | 72 | 15.6 | 82 | 15.9 |
| Province 2 | 78 | 16.9 | 184 | 35.7 |
| Province 3 | 83 | 18.2 | 63 | 12.1 |
| Province 4 | 57 | 12.4 | 26 | 5.1 |
| Province 5 | 102 | 22.1 | 80 | 15.4 |
| Province 6 | 28 | 5.9 | 40 | 7.9 |
| Province 7 | 40 | 8.9 | 40 | 7.9 |
| Season of childbirth |  |  |  |  |
| Winter | 139 | 30.2 | 112 | 21.8 |
| Spring | 120 | 26.1 | 146 | 28.3 |
| Summer | 100 | 21.7 | 149 | 28.9 |
| Autumn | 101 | 22.0 | 108 | 21.0 |
| ANC visits |  |  |  |  |
| Not visited | 15 | 3.4 | 23 | 4.6 |
| Visited once | 13 | 2.9 | 26 | 5.2 |
| Visited twice | 22 | 4.6 | 48 | 9.5 |
| Visited 3 times | 47 | 10.1 | 65 | 12.8 |
| Visited more than 3 times | 324 | 70.4 | 288 | 56.0 |
| Missing | 39 | 8.6 | 61 | 11.9 |

ANC-Antenatal care. The given sample size is adjusted to the survey sample weight.

**Supplementary Table 3. Immunization target and the coverage reported by Ministry of Health Nepal in 2016- 17.**

| **Vaccines** | **Target** | **Achieved** | **% Achieved** |
| --- | --- | --- | --- |
| BCG | 623929 | 569751 | 91.34 |
| OPV 1 | 623929 | 562452 | 90.14 |
| OPV 2 | 623929 | 550544 | 88.23 |
| OPV 3 | 623929 | 536191 | 85.93 |
| Penta 1 | 623929 | 566098 | 90.73 |
| Penta 2 | 623929 | 552190 | 88.50 |
| Penta 3 | 623929 | 539698 | 86.49 |
| MMR 1st dose | 623929 | 524332 | 84.03 |

Source: Annual Report, Department of Health Services 2016/17, Ministry of Health and Population, government of Nepal

**Supplementary material: Detail method of data collection in Nepal**

The Nepal Demographic and Health Survey 2016 (NDHS) is a nationally representative population survey conducted under the aegis of the Ministry of Health (MOH), Government of Nepal. It is the fifth NDHS series that provides up-to-date estimated of basic demographic and health indicators for Nepal and each ecological zone (Terai, Hills, and Mountains) and province (Province 1-7). The clinical, anthropometric and biochemical (CAB) component of NDHS 2016 provides information about hypertension through series of biomarker tests and measurements.

The 2016 NDHS used multistage stratified cluster sampling method to collect data from households nation-wide. The sampling frame used for 2016 NDHS was updated version of the frame from the 2011 National Population and Housing Census (NPHC). The 2016 NDHS included 263 municipalities and 7 provinces which were not in NPHC 2011). Samples were stratified and selected in two stages in rural areas and three stages in urban areas. The sampling frame contained information about ward location, type of residence (urban and rural), estimated number of residential households and estimated population. In rural areas, wards were small (average of 104 households) and served as the primary sampling units PSUs and households were selected from these PSUs. In urban areas, wards were large, with average of 800 households per ward. These wards were further segmented into small enumeration areas (EAs). Selected EAs served as PSUs and households were selected from them.

In the first stage, 383 wards were selected with probability proportional to ward size and with independent selection in each sampling stratum. Due to large size of the urban wards, in a second stage of sample selection, on EA was randomly selected from each of the sample urban ward. A household listing was done in all the selected sampling clusters (rural wards and urban EAs), and the resulting lists of households served as the sampling frame for the selection of households in the next stage. Some of the large clusters with more than 200 households were segmented further to minimize the task of household listing. In last stage of selection, a fixed number of 30 households per cluster were selected with an equal probability systematic selection.

Out of total population 28.98 million in Nepal, 14,701 individuals (8,435 women and 6,266 men) from 11,040 households (6,978 in urban and 4,062 in rural) were selected with overall response rate of 96% (97% women and 95% men).
